# A squalamine derivative, NV669, as a novel PTP1B inhibitor: *in vitro* and *in vivo* effects on pancreatic and hepatic tumor growth

**DOI:** 10.18632/oncotarget.27286

**Published:** 2019-11-19

**Authors:** Sylvie Carmona, Jean-Michel Brunel, Rénaté Bonier, Véronique Sbarra, Stéphane Robert, Patrick Borentain, Dominique Lombardo, Eric Mas, René Gerolami

**Affiliations:** ^1^Aix Marseille Univ, INSERM, CRO2, Centre de Recherche en Oncologie biologique et Oncopharmacologie, Faculté de médecine, Marseille, France; ^2^Aix Marseille Univ, CNRS, INP, Institut de Neuro-Physiopathologie, Faculté de médecine, Marseille, France; ^3^Aix Marseille Univ, INSERM, SSA, MCT, Marseille, France; ^4^Aix-Marseille Univ, CNRS, INSERM, Institut Paoli-Calmettes, CRCM, Centre de Recherche en Cancérologie de Marseille, Marseille, France; ^5^Aix Marseille Univ, INSERM, INRA, C2VN, Faculté de médecine, Marseille, France; ^6^Aix Marseille Univ, INSERM, INRA, C2VN AMUTICYT Core facility, Faculté de pharmacie, Marseille, France; ^7^Aix Marseille Univ, AP-HM, Assistance Publique des Hôpitaux de Marseille, Centre Hospitalo-Universitaire Timone, Service d’Hépato-Gastro-Entérologie, Marseille, France

**Keywords:** aminosterol, pancreas, liver, PTP1B, cancer

## Abstract

NV669 is an aminosterol derived from squalamine found to possess strong anticancer effects. The aim of this study was to investigate NV669’s beneficial effects on human pancreatic and hepatic cancer models and to decipher the cellular and molecular mechanisms involved in tumor growth decrease upon treatment with NV669.

Pancreatic (BxPC3, MiaPaCa-2) and hepatic (HepG2, Huh7) cancer cells were treated with NV669, and the effects recorded on proliferation, cell cycle and death. Results showed that NV669 inhibited the viability of cancer cells, induced cell cycle arrest and subsequently promoted apoptosis. This was accompanied by a decrease in the expression of cyclin B1 and phosphorylated Cdk1 and by a cleavage of pro-apoptotic caspase-8 and PARP-1. Taken together, our studies showed that NV669 inhibits the proliferation of pancreatic and hepatic cancer cells through the regulation of G2/M phase transition *via* the cyclin B1-Cdk1 complex.

*In vitro* NV669 inhibits PTP1B activity and FAK expression. NV669 impacts on the expression of adhesion molecules CDH-1, -2 and -3 in BxPC3 and Huh7 lines that form cell monolayers. Consecutively NV669 induces cell detachment. This suggests that NV669 by inhibiting PTP1B induces cell detachment and apoptosis.

Subsequently, our *in vivo* results showed that NV669 inhibited the growth of pancreatic and hepatic tumor xenografts with a significant cell cycle arrest in pre-mitotic phase and an increase of tumor cell apoptosis. Therefore, NV669 may serve as an alternative anticancer agent, used alone or in association with other medications, for the treatment of pancreatic adenocarcinoma and hepatocellular carcinoma.

## INTRODUCTION

Pancreatic ductal adenocarcinoma (PDAC) and hepatocellular carcinoma (HCC) are common solid organ malignancies worldwide (https://www.cancer.org/content/dam/cancer-org/research/cancer-facts-and-statistics/annual-cancer-facts-and-figures/2018/cancer-facts-and-figures-2018.pdf) [1]. Surgical resection is the only curative therapy but can be proposed only to a small percentage of patients with small tumors. Patients with unresectable tumor have a dismal prognostic. Indeed, the benefits of systemic therapies such as gemcitabine (cytosine analogue) and sorafenib (multikinase inhibitor), respectively for pancreatic and liver cancer, remain low in these patients [2–4]. As a result, the search for more effective chemotherapeutic agents is still ongoing.

Previously, many studies have examined the antitumoral effect of squalamine, a natural aminosterol, initially isolated from the liver of the shark *Squalus acanthias* [5]. Squalamine is now chemically synthesized [6] for it clinical applications and known to have a strong anti-angiogenic activity *in vitro* and *in vivo* [7, 8]. Hence, the antiangiogenic activity of squalamine was confirmed in various tumor xenograft models. Squalamine efficiently inhibited the growth of tumors of lung, breast, brain, ovaries and prostate implanted in nude mice [9–13]. Squalamine was also assessed in phases I and II of clinical trials on lung cancer [14, 15]. The way of squalamine cell capture and the intracellular signalling pathways activated by this drug remain unclear. Albeit squalamine is a steroid, it does not interact with the receptors of glucocorticoids [16]. However, it is suggested that it could interact with NHE-3 exchanger [17]. In this study we synthesized squalamine analogues with the expectation to obtain a more efficacious derivative.

We report herein the design of new aminosteroid derivatives easily obtained from cheap and available precursors through an original titanium reductive amination reaction [18, 19]. Further we report the anticancer activities of a new polyaminosteroid derivative, referred to as NV669, and a deeper analysis of its mechanism of action pointing out its originality to fight cancer.

Data showed that NV669 potently inhibits PDAC and HCC cell proliferation, induces a pre-mitotic cell cycle arrest and promotes apoptosis both *in* *vitro* and *in vivo*. We suggested that NV669 acts on tumor growth by blocking the PTP1B phosphatase activity and inducing cell death by detachment.

## RESULTS

### Synthesis of NV669

**Scheme 1:**
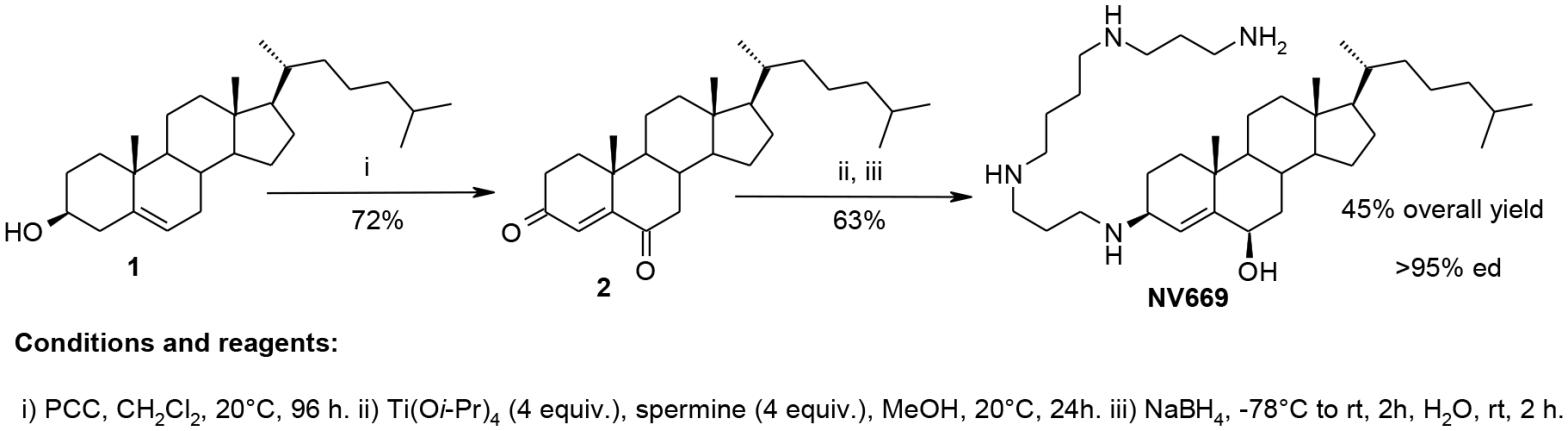
Synthesis of NV669

By using an efficient titanium reductive amination reaction [18, 19], we have envisioned in a two steps synthesis procedure the preparation of a new polyaminosteroid derivative from cholesterol **1** available in large amounts. The synthetic pathway is given in the following scheme 1. The expected polyaminosteroid derivative NV669 was obtained in a 45% overall yield with an excellent diastereoselectivity up to 95% involving the major formation of the 3β, 6β diastereomer (Scheme 1). NV669 is > 95% pure and stored in distilled water.

### NV669 inhibits pancreatic and hepatic cancer cell proliferation

**Figure 1:**
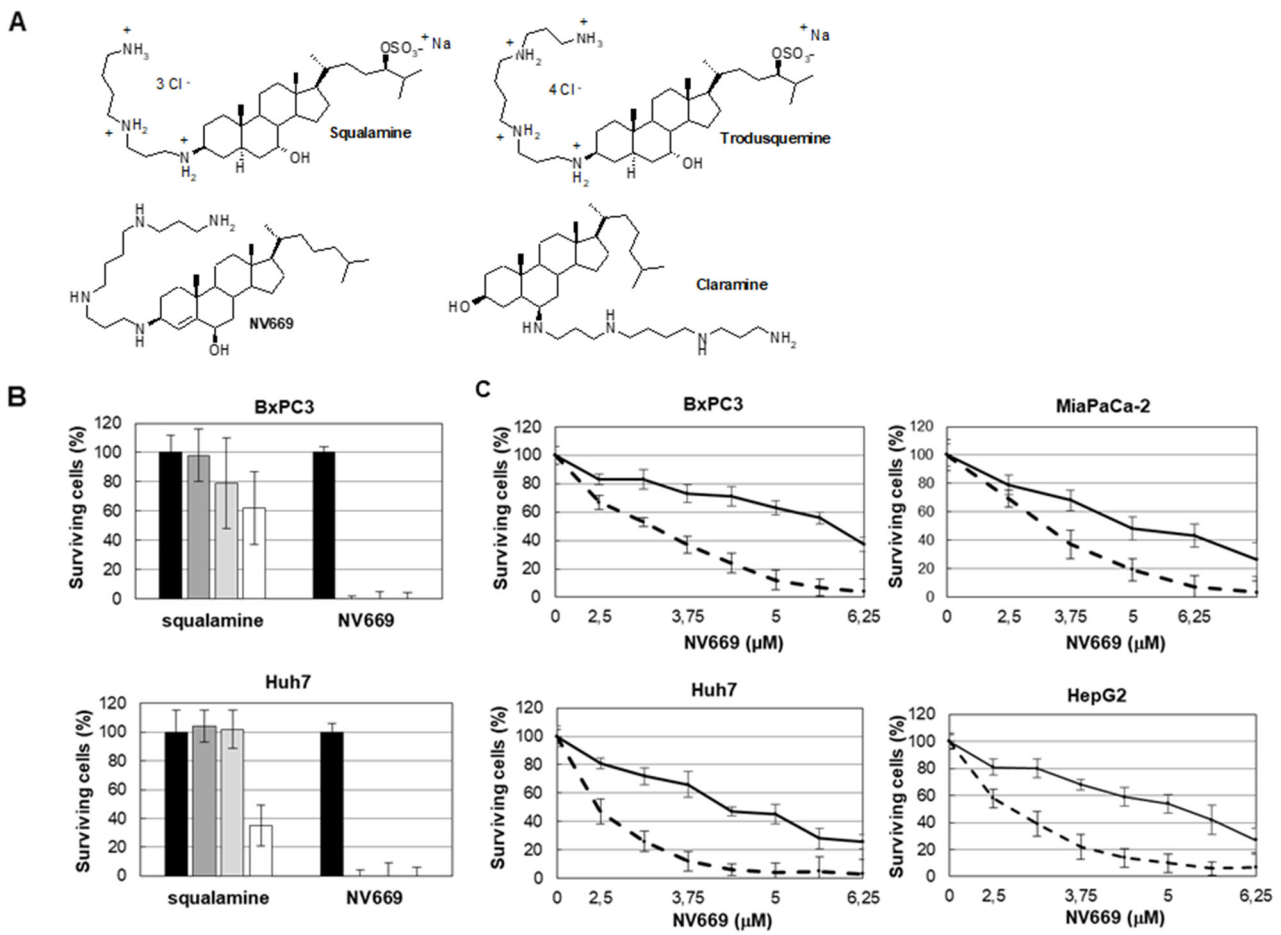
NV669 inhibited pancreatic and liver cancer cell proliferation **(A)** The chemical structures of squalamine (7, 24 dihydroxylated 24-sulfated cholestane steroïd conjugated to a spermidine at C-3 top left), trodusquemine (top right), NV669 (bottom left) and claramine (bottom right). **(B)** BxPC3 and Huh7 cells were treated 72h with squalamine or NV669 at indicated concentrations. The cell viability was then assessed by the crystal violet assay. Black column, 0 μM; dark-grey column, 10 μM; grey column, 50 μM and white column, 100 μM. Values are means +/- SD of at least three independent experiments. **(C)** Effects of NV669 treatment on proliferation of BxPC3, MiaPaCa-2, HepG2 and Huh7. Cells were incubated with various doses of NV669 for 24h (full line) and 72h (dotted line) then analysed for cell proliferation using colorimetric dosage. Values are means +/- SD of at least six independent experiments.

Initially, we investigated the effects of NV669, a derivative of squalamine (Figure 1A) on cell proliferation of human pancreatic (BxPC3, MiaPaca-2) and hepatic (HepG2, Huh7) cell lines. BxPC3 and MiaPaca-2 have p53 mutation but BxPC3 is a differentiated cell type and MiaPaca-2 cell is considered as a cancer stem-like cell resistant to curative drugs [20]. HepG2 has wild type p53 whereas in Huh7 p53 is mutated. Our results showed that NV669 inhibited significantly the viability of all cancer cell lines in a dose- and time-dependent manner (Figure 1C). The concentration which leads to 50 % of hepatic and pancreatic cell death (IC_50_) is close to 5 μM after 24 h and 3 μM after 72 h (Table I). Squalamine [21] decreased the cell proliferation but much less efficacious than NV669 (Figure 1B).

**Table 1:** NV669 IC_50_ (μM) on pancreatic and hepatic cell lines

	24 h	72 h
BxPc-3	5.61 ± 1.02	3.21 ± 0.42
MiaPaCa-2	5.05 ± 1.12	3.14 ± 0.62
HepG2	4.82 ± 0.98	2.67 ± 0.26
Huh-7	4.60 ± 0.41	2.41 ± 0.28

Cells were seeded onto 96-well plates at a density of 1.5×10^3^ cells per well for BxPC3 cells, 0.5×10^3^ cells/well for MiaPaCa-2 cells, 3×10^3^ cells/well for HepG2 cells and 0.85×10^3^cells/well for Huh7 cells and allowed to grow overnight in complete media. The cells were then treated with increasing doses of NV669 for 24 and 72 hours. Growth inhibition of cells was recorded by using the crystal violet staining assay. The concentrations of NV669 that inhibited 50 % of cell growth (IC_50_) were graphically determined. Values are average of at least six independent experiments and expressed as mean +/- standard deviation.

### NV669 induces G2/M cell cycle arrest in pancreatic and hepatic cancer cells

**Figure 2:**
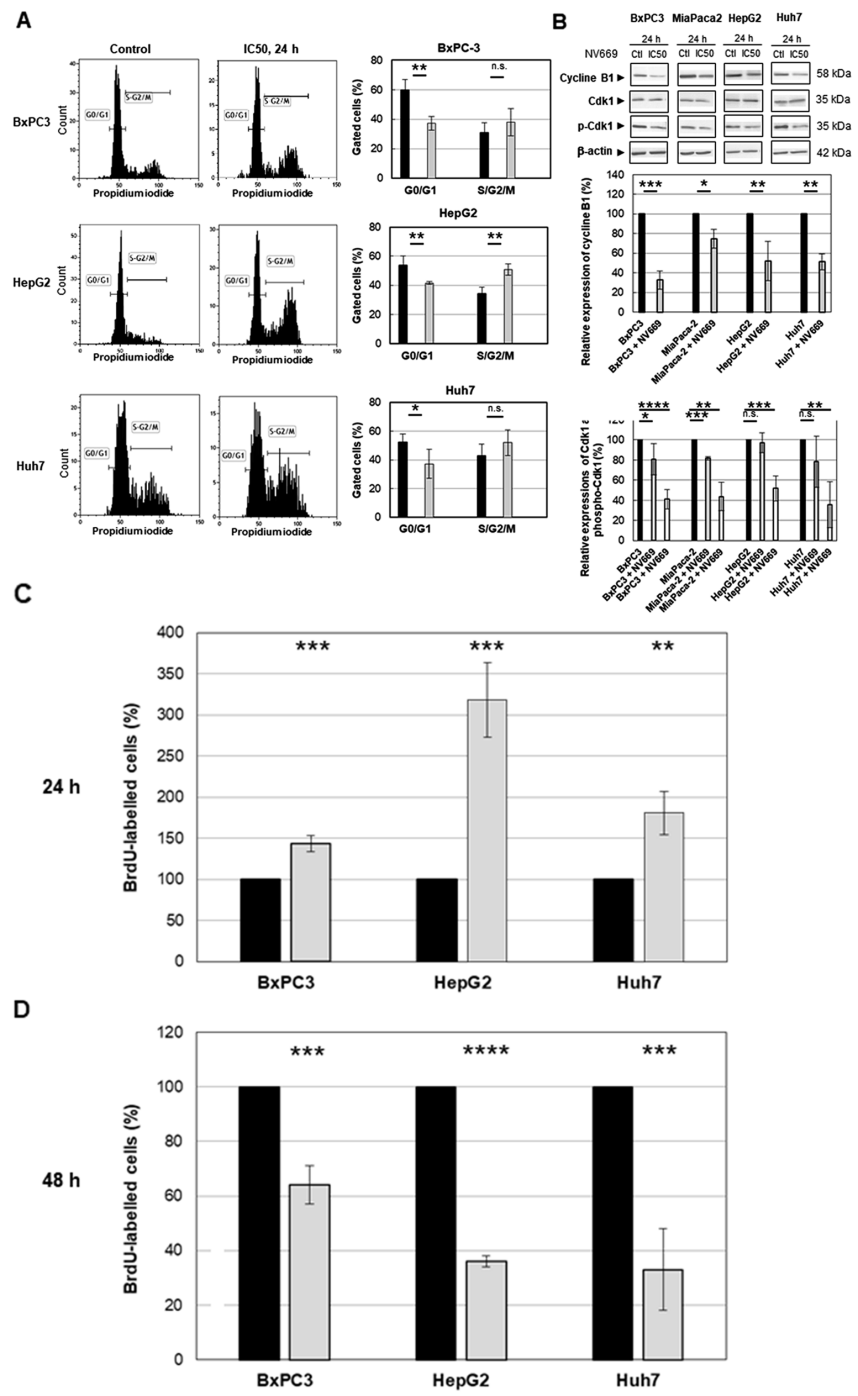
NV669 induced cell cycle arrest **(A)** DNA content and cell cycle analysis of NV669-treated cells. The three cells lines BxPC3, HepG2 and Huh7 were incubated with NV669 at the IC_50_ dose for 24 h. The number of cells in G0/G1 (M1) and S/G2/M phases were determined by flow cytometry as described in Material and Methods. Black column, control and grey column, NV669 treated cells. **(B)** Expression of cyclin B1 determined by western blots (upper panel). Western blot were quantitated for cyclin B1 expression (central panel, black column, control; grey column NV669-treated cells), for Cdk1 expression, (lower panel, black column, control; grey column NV669-treated cells) and for Tyr15 phosphorylation of Cdk1 (phospho-Cdk1) (lower panel, black column, control; white column NV669-treated cells). Quantifications were determined after treatment with IC_50_ concentration of NV669 (see table 1) for 24 h. β-actin was used as internal control. **(C, D)** Cells were pulsed with 10 μM BrdU for 2 hours before drug addition and their cell cycle distribution was observed after a 24 h treatment by NV669 at IC_50_ (C). On the other hand, cells were treated with IC_50_ of NV669 for 24 h then stained with BrdU for 2 hours. Cells with a 4n DNA content were quantified 24 h later (D). A colorimetric immunoassay allows to determinate the percentage of BrdU-labelled cells resulting from their progression through the S phase in control cells (black columns) and in NV669-treated cells (grey columns). Values are means +/- SD of at least three independent experiments ^****^, p<0.0001; ^***^, p<0.001; ^**^, p<0.01; ^*^, p<0.5; n.s. not significant.

To determine whether the growth inhibition of pancreatic and hepatic cancer cells by NV669 was caused by cell cycle arrest, the cells were treated with IC_50_ concentration of NV669 for 24 h. The cells were then fixed, incubated with RNase A and stained with propidium iodide. Two cell cycle populations, G0/G1 and S/G2/M were determined by flow cytometry. The percentage of BxPC3, HepG2 and Huh7 cells decreased at the G0/G1 phase while it increased, significantly for HepG2 cells, at S/G2/M phase. Thus, independently of the cell model these data showed a decrease in cell population in G0/G1 and a rise of cell population in G2/M transition (Figure 2A).

Subsequently, western blot analyses indicated that the expression of G2/M cell cycle regulating factor cyclin B1 has decreased significantly after treatment with IC_50_ concentration of NV669 for 24 h (Figure 2B). Compared to the expression of Cdk1 (cdc2), that of phosphorylated Cdk1 (p-Cdk1) decreases significantly upon NV669 treatment at IC_50_ for 24h (Figure 2B). These results suggest that the inhibition of cell proliferation by NV669 is associated with the induction of G2/M phase arrest.

### NV669 induces a pre-mitotic cell cycle arrest.

The cell cycle results raised the possibility that the increase in the amount of cells in phase S/G2/M by NV669 would be consistent with an arrest in G2/M or with an increased proliferation, with cells driven from G0/G1 phase to S/G2/M phase. To confirm that the 4n cells present after NV669 treatment resulted from pre-mitotic arrest rather the progression of 4n cells through mitosis to the next G1 phase, we pulsed cells with 10 μM BrdU for 2 hours before or after NV669 treatment at IC_50_ for 24h. Then, we determined the percentage of labelled S phase cells that incorporated BrdU during DNA synthesis (Figure 2C and 2D). Results indicated that when we added BrdU in cell cultures before NV669 treatment (Figure 2C), cells in S phase accumulate in a 4n state (143.5 ± 10 % for BxPC3, 318.5 ± 45 % for HepG2 and 181 ± 26 % for Huh7) compared with control cells. In addition, when cells were pulsed with BrdU after 24h treatment by NV669 (Figure 2D), the percentage of BrdU-labelled cells strongly decreased with the treatment at IC_50_ concentration of NV669 (64±7 % for BxPC3, 36± 2% for HepG2 and 33±15 % for Huh7) compared with control cells. These results suggest that NV669 might cause the cells to arrest in G2 before they ever reach mitosis.

### NV669 induces apoptosis in pancreatic and hepatic cancer cells

**Figure 3:**
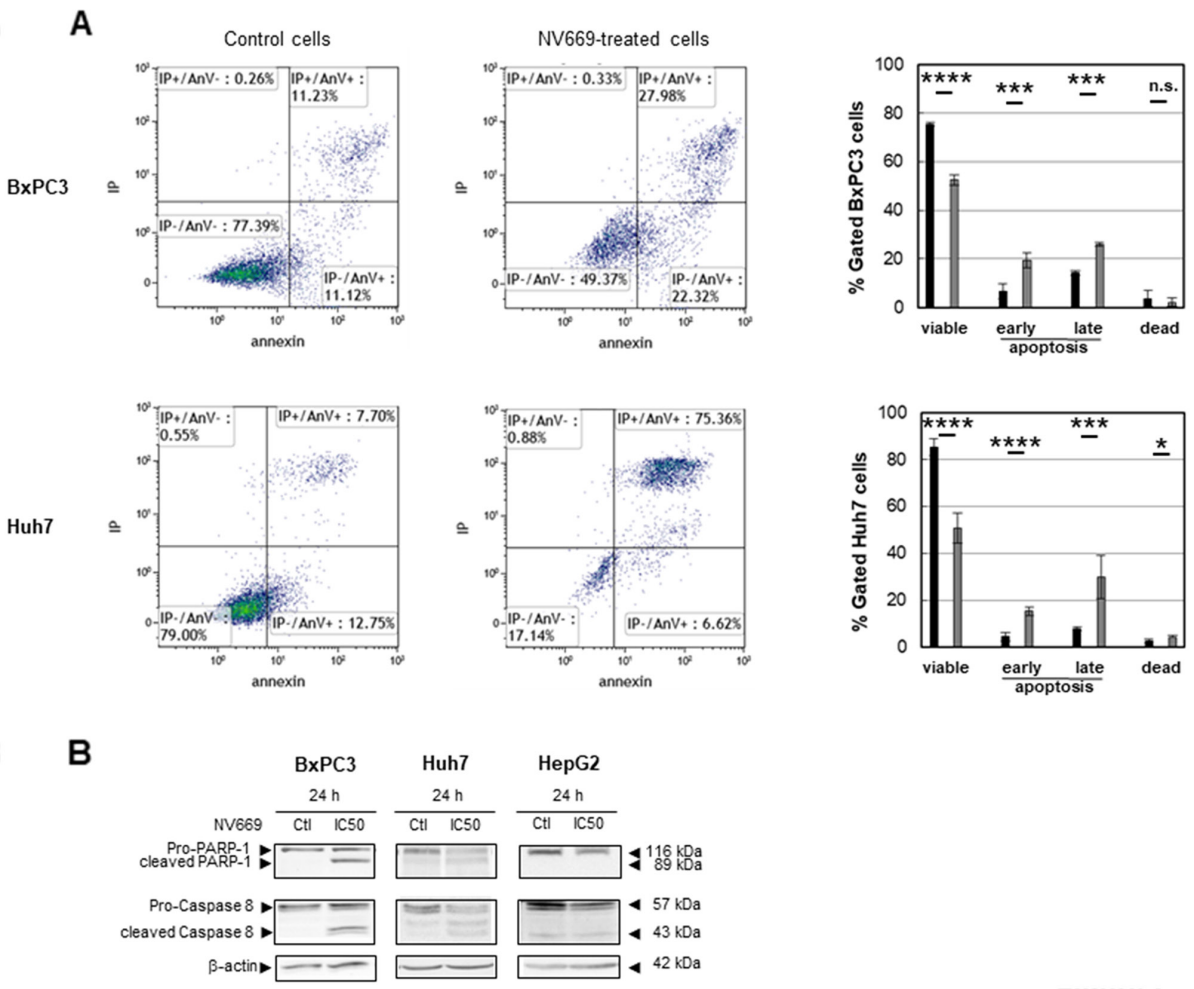
NV669 induced cancer cell apoptosis BxPC3 and Huh7 cancer cells lines were treated with IC_50_ concentration of NV669 for 24 h. **(A)** Apoptosis levels were detected by flow cytometry analysis using annexin V-FITC/propidium iodide double labelling of cells either incubated without (left panels) or with (central panels) NV669 (data of one experiment of three is shown). Quantifications are shown in the right panels (black column, control; grey column NV669-treated cells). Values are means +/- SD of three independent experiments ^****^, p<0.0001; ^***^, p<0.001; ^**^, p<0.01; ^*^, p<0.5; n.s. not significant. **(B)** Target proteins, total Caspase-8 and PARP-1, were detected by western blot analyses.

To further research whether the decrease of cell proliferation by NV669 observed on pancreatic and hepatic cells resulted from the cell death by apoptosis, flow cytometry was carried out after annexin V/propidium iodide double labelling of cells. After treatment with IC_50_ concentration of NV669 for 24 h, the percentage of cells at early (annexin-V positive cells, propidium iodide negative cells) and late (annexin-V positive cells, propidium iodide positive cells) stages of apoptosis significantly increased from control to NV669-treated cells, independently of the tumor cell origin (Figure 3A). Viable cells surviving to treatment were not stained. These results revealed that NV669 may induce death through apoptosis. The expression of poly (ADP-ribose) polymerase (PARP) and caspases were also examined. Our results showed a cleavage of caspase-8, PARP-1 (Figure 3B) and nuclear envelop proteins lamins (data not shown). These data strongly suggested that NV669 induced the apoptosis of pancreatic and hepatic cancer cells by activating caspase-8 and promoting PARP-1 cleavage.

### NV669 inhibits *in vitro* PTP-1B activity

**Figure 4:**
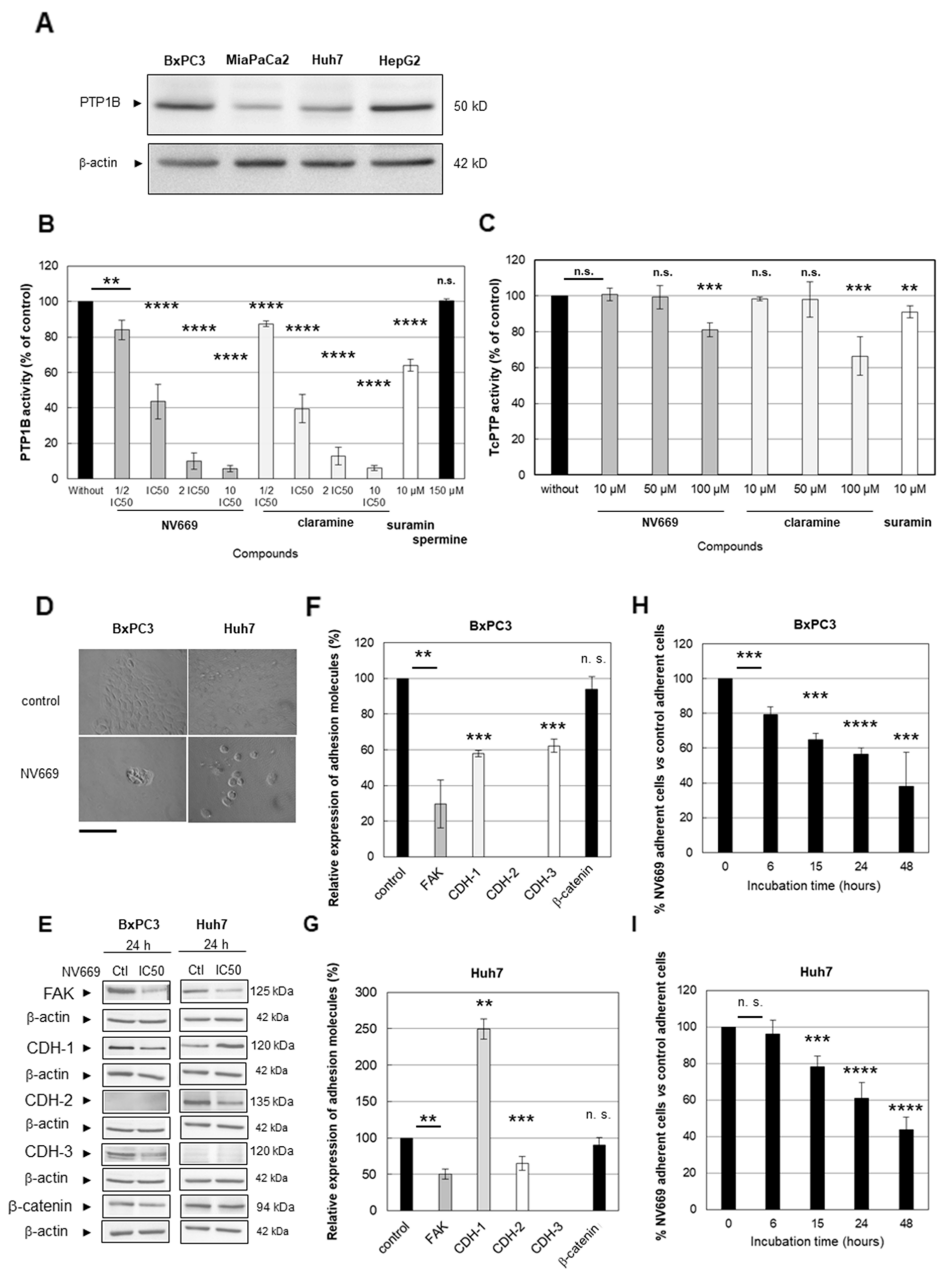
NV669 affected the expression of cell adhesion molecules and induced cell detachment **(A)** Expression of PTP1B in BxPC3, MiaPaCa-2, HepG2 and Huh7 cancer cells lines. **(B)** Recombinant human PTP1B or **(C)** Tc-PTP were incubated in a microplate with 75 μM of phosphopeptide IR5 insulin receptor β-subunit domain and with increasing doses of NV669 (dark grey columns) or claramine (light grey columns), for 30 min at 30°C. Cells were also incubated with suramin (10 μM, white column) as positive control or spermine (150 μM, black column on the right) as negative control, for 30 min at 30°C. A colorimetric assay allows to determinate the released phosphate during enzymatic reactions. Values are means +/- SD of three triplicate experiments per conditions. **(D)** Cell morphology change after NV669 treatment was assessed using phase-contrast microscope (scale bar = 250 μm). **(E)** Expression of FAK, cadherin-1 (CDH-1), cadherin-2 (CDH-2), cadherin-3 (CDH-3) and β-catenin is determined by western blot after treatment with IC_50_ concentration of NV669 for 24 h. β-actin was used as loading control. **(F, G)** The relative expression of these proteins was calculated by Image J software for BxPC3 (F) and Huh7 (G) cells. Values correspond to band intensity reported to actin band intensity and expressed as % of control values (without NV669 treatment). **(H, I)** Effects of NV669 at IC_50_, 24h on cell detachment. Values are means +/- SD of three independent experiments. ^****^, p<0.0001; ^***^, p<0.001; ^**^, p<0.01 and ^*^, p<0.5; n.s, not significant.

Previous report demonstrated that the aminosterol claramine – and its analogue trodusquemine – two steroid-spermine conjugates, could activate components of insulin signalling by targeting the protein tyrosine phosphatase 1B (PTP1B) [22]. Hence, we investigated whether the effect of NV669 on cancer cells is associated with the inhibition of PTP1B activity. Firstly, we showed that PTP1B phosphatase is effectively expressed by hepatic and pancreatic cells used in the present study (Figure 4A). We then carried out *in vitro* colorimetric assays on recombinant human PTP1B and T-cell protein tyrosine phosphatase (Tc-PTP). Like claramine (a PTP1B inhibitor used here as positive control), we found that NV669 blocked significantly *in vitro* PTP1B activity in a dose- and time-dependent manner (Figure 4B). NV669 and claramine have no effect on Tc-PTP activity (Figure 4C). Therefore, NV669 inhibits PTP1B but not its closest related phosphatase Tc-PTP. By contrast spermine, the poly-amino structure of which is that of the side chain of claramine and trodusquemine, had effect neither on PTP1B activity (Figure 4B), nor on Tc-PTP activity (data not shown). The PTP1B inhibitor suramin [23] supplied in the PTP1B colorimetric assay kit used here effectively inhibits the PTP1B activity but has a poor effect on Tc-PTP activity (Figure 4B, 4C).

### NV669 impacts on the expression of cell adhesion molecules and induces cell detachment.

A recent study by Hilmarsdotti *et al.* [24] shows that PTB1B inhibition affects the expression of adhesion molecules and consequently induces apoptosis by cell detachment (anoikis). Treatment of BxPC3 pancreatic and Huh-7 hepatic cancer cells with NV669 at IC_50_ for 24h results in a failure to establish a confluent monolayer (Figure 4D) with cells forming rounded “grape-like” structures or isolated cells. This is less probing with MiaPaCa-2 and HepG2 cells which do not form a confluent monolayer even in absence of NV669 (data not shown). To further confirm that NV669 affects cell adhesion we treated BxPC3 and Huh7 cell models with NV669 in the conditions described above and tested the expression of selected adhesion molecules. As shown in Figures 4E, 4F and 4G cell treatment with NV669 significantly reduced the expression of Focal Adhesion Kinase (FAK), a key-molecule in cell-matrix adhesion. The expression of cadherins which are involved in cell-to-cell contact is also significantly affected upon NV669 treatment of cell models used herein. In BxPC3 cells both cadherin-1 and -3 expressions were significantly decreased while in Huh7 cells cadherin-2 expression was shifted to cadherin-1 expression (Figure 4E, 4F and 4G). Independently of the cell model used β-catenin expression remained unchanged (Figure 4E, 4F and 4G). Taken all together, these data strongly suggested that the cell-to-cell contact is modulated by NV669. Therefore we investigated cell detachment upon incubation of cell monolayer with IC_50_ of NV669. As shown on Figure 4H and 4I, a significant decrease in adherent cell with time beginning after 6h or 15h incubation occurs respectively, when BxPC-3 and Huh-7 cells were treated with NV669. Consequently apoptosis may happen consecutive to a decrease in the adherence potential of the cell. This result agrees with the modification in the expression of adherence molecules (see Figure 4E).

### NV669 inhibits *in vivo* tumor growth

**Figure 5:**
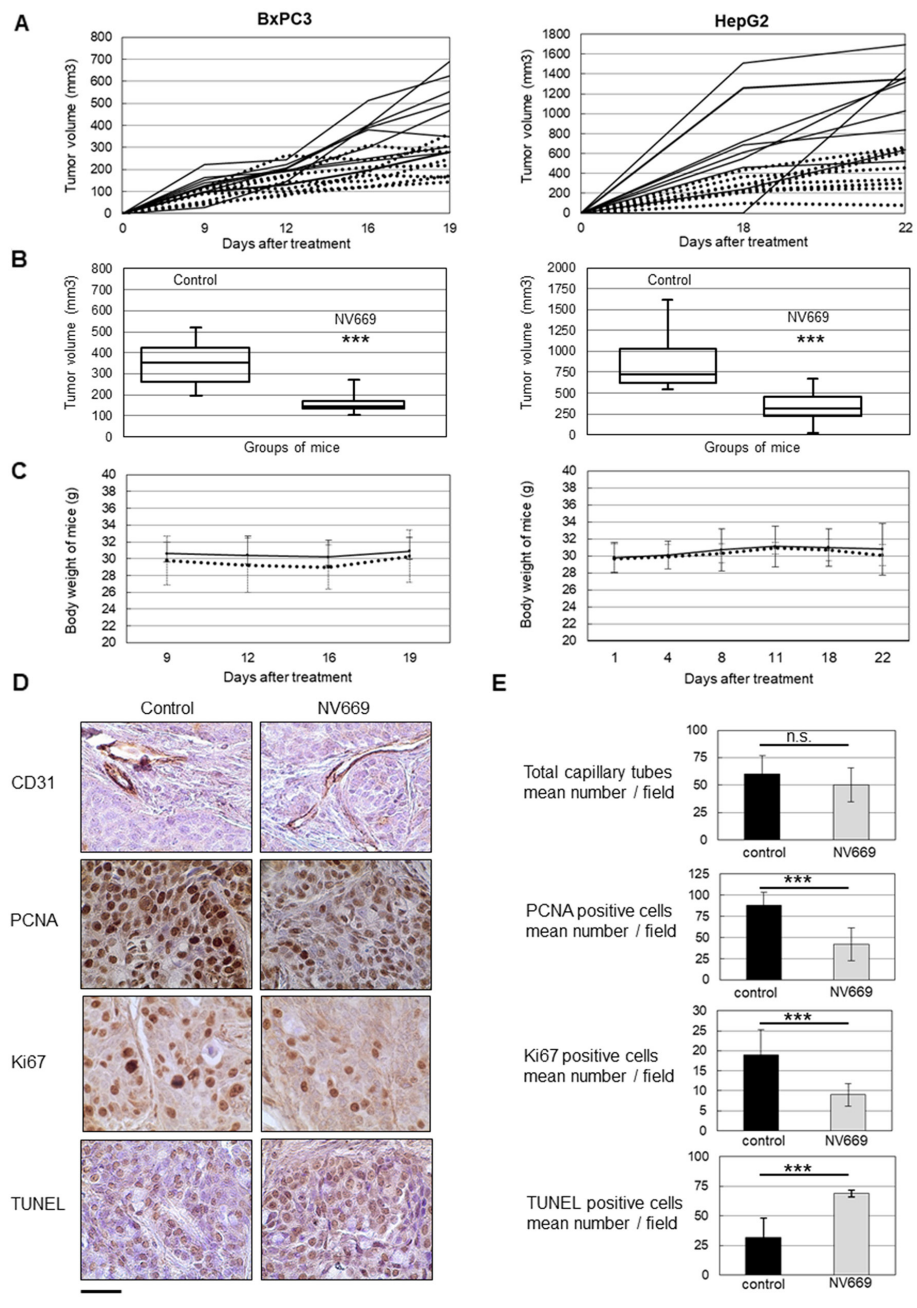
NV669 inhibited pancreatic and liver cancer tumor xenograft growth and induced apoptosis, *in vivo* BxPC3 (5 × 10^6^) cells or HepG2 (10 × 10^6^) cells were injected in subcutaneous to the flanks of nude mice. At the same time, 4 mg/kg/d of NV669 was injected daily sc; for up to 20-22 days. **(A)** The tumor growth of NV669 treated mice (dotted line) and of untreated mice (solid line) was monitored daily for 20-22 days. **(B)** Mice were sacrificed on day 21-23 and tumors were collected. Box-plot represents tumor volume at sacrifice of NV669-treated mice (NV699) or untreated mice (control). (^***^, p < 0.001). Reduction in tumor volume from NV669-treated animals was compared with untreated controls. **(C)** Body weight of the nude mice in NV669 treated (dotted line) and control groups (solid line). **(D)** Tumor sections from NV669-treated mice (NV699) or untreated mice (control) were stained with an anti-CD31, anti-PCNA and anti-Ki-67 antibodies, or TUNEL agent (scale bar = 75 μm). **(E)** The number of CD31 positive capillary tubes, PCNA/Ki67 expressing cells and TUNEL positive cells was assessed in tumor sections by Image J software (https://imagej.nih.gov/ij/), to evaluate the impact of NV669 on angiogenesis, cell proliferation and apoptosis, respectively. Assay was done in multiplicate and values are means +/- SD of three independent experiments. ^***^, p<0.001; n.s, not significant.

Relapses are frequently observed in PDAC – the diagnosis of which is always too late due to the absence of specific symptoms - and HCC within few years following surgical resection. Therefore we next investigated the effects of NV669 on the establishment of pancreatic and hepatic tumors in murine models. For this purpose BxPC3 or HepG2 cells were xenografted subcutaneously (sc.) in nude mice. At the same time, 4 mg/kg of mouse/day NV669 was given sc. once a day for 3 weeks. Results showed that, over time, the majority of tumors in NV669 treated mice growth slower than those in untreated control mice (Figure 5A). At the end of experiments, tumors were excised from animals. 75 % of tumors from NV669-treated mice have significantly lower volumes than overall control tumors (Figure 5B). Moreover, there was no cytotoxicity of NV669 as shown by the lack of changes in animal body weight and behavior during the time of experiments (Figure 5C).

### NV669 induces *in vivo* a decrease of proliferation and an increase in apoptosis

To determine whether anti-tumor effect of NV669 results from an anti-angiogenic effect, an anti-proliferative or a pro-apoptotic effect, an immunohistochemical analysis was performed on excised BxPC3 tumors. Sections of fixed tumors were stained with antibodies raised to CD31 to highlight angiogenesis, to PCNA and Ki67 to detect proliferating cells or stained with TUNEL agent to visualize apoptotic cells (Figure 5D). CD31 staining showed no significant difference between the number of capillary tubes in tumors from mice treated with NV669 and that from control mice. There was no significant difference in diameters of blood vessels also (data not shown). By contrast, PCNA and Ki67 staining showed that the number of Ki67 positive tumor cells was substantially less in tumors of NV669-treated mice, when compared with control tumors. Tumor xenografts from NV669-treated group exhibited a marked rise in TUNEL-positive cells compared to the control group. Quantification of stained samples indicated two-fold decrease in the number of PCNA or Ki67-positive cells (p < 10^-3^) and two-fold increase in the number of TUNEL-positive cells (p < 10^-3^) in the NV669-treated group compared to the control group (Figure 5E).

## DISCUSSION

PDAC and HCC are major causes of cancer death worldwide. Most patients have inoperable diseases at the time of diagnosis and need systemic therapy. Chemotherapy agents have shown low benefit in controlled clinical trials and treatment outcome remained poor due to drug resistance and toxicity. Relapses are frequently observed in the years following surgery. In the current study, results show that NV669, a derivative of squalamine, potently inhibits the proliferation of pancreatic and hepatic cancer cells, induces cell cycle arrest in G2/M pre-mitotic phase and apoptosis both *in vitro* and *in vivo*. Besides, we report a mechanism by which NV669 decreases the proliferation of pancreatic and hepatic cancer cells likely through the inhibition of PTP1B activity, resulting in the modification of cell-to-cell contact and cell adherence ending with cell detachment and apoptosis.

The results of our *in vitro* studies have demonstrated that NV669 inhibits proliferation of cancer cells in a dose- and time-dependent manner. On the other hand, squalamine has no significant effect on studied pancreatic and hepatic tumor cells. Indeed, investigations of Li *et al.* have already showed that squalamine does not affect tumor cells in culture [21]. These results suggest that the nature (and stereochemistry) of chemical groups linked to the sterol backbone plays an important role in their cytotoxic activities. Because PDAC and HCC, like many other types of cancer, are characterized by accelerated cell proliferation and by resistance to death stimuli, we have examined the cytotoxic effect of NV669 on programmed cell death/apoptosis and cell cycle.

In the present study, we found that NV669-mediated mitotic block was achieved *via* a decrease in cyclin B1 and in phosphorylated Cdk1 expressions. Consequently, the NV669 cytotoxic effect on pancreatic and hepatic cancer cells is likely due to a cell cycle arrest at G2/M phase. Cyclins play pivotal roles at various phases of the cell cycle by activating specific cyclin-dependent-kinases (Cdk). Cyclin B1, encoded by the CCNB1 gene, is the regulatory subunit of Cdk1 (which is also called cdc2). The cyclin B1/Cdk1 complex controls the G2 phase and is critical for the initiation of mitosis [25–27]. Clinical studies have reported an overexpression of cyclin B1 in various human cancers [28–36]. Elevated expression of cyclin B1 is correlated with poor survival in most solid tumors [37–46]. Furthermore, overexpression of cyclin B1 was associated with high histological grade of differentiation and vascular invasion [30, 42]. Finally, the uncontrolled expression of cyclin B1 is an early event in the transformation process [47, 48]. The abnormally expressed cyclin B1 is also considered as a tumor antigen and used for early cancer detection [49–52]. Previous studies have shown that downregulation of cyclin B1 specifically suppresses the activity of Cdk1 further leading to a strong proliferation inhibition and apoptosis induction in various cancer cells, both *in vitro* and *in vivo* [53, 54]. A prominent downstream target of the cyclin B1/Cdk1 is the tumor suppressor p53 [55]. The induction of apoptosis by cyclin B1/Cdk1 inhibition depends on functional p53 signaling [56]. NV669 inhibited pancreatic and hepatic cell models independently of their wild-type (HepG2) or mutated (Huh-7, BxPC-3 and MiaPaCa-2) p53 status suggesting that the NV669 effect on cell growth is independent of p53.

Initiator caspase-8 and caspase-9 involved in the apoptotic cascade are important modulators of the response to cyclin B1/Cdk1 inhibition [57–60]. The relationship between cell cycle and apoptosis is now evident [61]. Hence, we examined whether the cell growth inhibition observed upon NV669 treatment was associated with increased apoptosis. BxPC3, HepG2 and Huh7 cells lines treated with NV669 displayed typical apoptotic morphological changes, including cell shrinkage, nuclear and DNA fragmentation and chromatin condensation (data not shown). Apoptotic cell death was demonstrated by FACS analysis in liver and pancreatic tumor cells exposed to NV669. Finally we observed cleavage of caspase-8 and PARP-1 in NV669-treated cells. Thus, a potential mechanism for NV669-induced cell growth decrease is apoptosis.

The results of our *in vivo* studies have shown marked decrease of BxPC3 and HepG2 tumor growth in nude mice xenografts upon NV669 treatment. There was a significant reduction in tumor volume in NV669-treated animals compared with untreated controls without noticeable toxic effects. In excised tumors, we observed a significant decrease of tumor cell proliferation as shown by the results of Ki-67 and PCNA staining. These two nuclear proteins are expressed during cellular proliferation, Ki-67 expression during the mitosis phase [62, 63] while PCNA expression has been observed in S phase [64]. We also found in excised tumors from NV669-treated animals an increased number of apoptotic tumor cells observed by TUNEL immunostaining. We did not observe any modification in CD-31 staining in treated tumors suggesting that NV669 might not affect tumor angiogenesis *in vivo*.

Altogether, the antitumoral effect of NV669 is very different from that of squalamine, from which NV669 is derived. Indeed, several investigations have found that the antitumoral effect of squalamine was mainly related to its antiangiogenic properties. Indeed, squalamine blocks proliferation of HUVEC *in vitro* [7], prevents growth of new blood vessels in chick chorioallantoic membrane [6] and rabbit cornea [8] and impairs neoangiogenesis in xenografts tumors in mice originating from different types of cancer [9–13].

Based on these results, we searched for a target of NV669. Our results indicate that NV669 specifically inhibits PTP1B *in vitro*. PTP1B is a non-transmembrane protein tyrosine phosphatase [65, 66] which regulates metabolism, inhibiting insulin and leptin signaling [67]. Recently, Qin *et al.* showed that two aminosterols, claramine and trodusquemine, activated insulin signaling upon PTP1B inhibition in cultured neuronal cells [22]. Moreover, there are founded suspicions that PTP1B plays a role in tumorigenesis. PTP1B stimulates cell proliferation mediated by Src and Ras in human breast and liver cancer murine models [68–71]. It has also been shown that increased PTP1B activity could decrease the antitumoral effect of sorafenib in primary liver cancer therapy [72]. NV669 by decreasing PTP1B activity could therefore be a candidate to restore sorafenib antitumoral activity. Further studies are necessary to address the potential efficacy of the association of NV669 and sorafenib in liver tumor cells.

It is well known that tumor progression involves changes in tumor cell capacities to adhere and communicate with neighboring cells. Changes in cadherin expression have a critical role in tumor progression and metastasis. In this study we observed that NV669 promotes changes in the expression levels of cadherins. Indeed, cadherin-2 and cadherin-3 expressions, which are associated with malignant clinicopathological characteristics, respectively in hepatocellular carcinoma and pancreatic cancer [73, 74] are downregulated. By changing cadherin expression and modifying cell-cell contacts, NV669 could induce cell rounding and possibly anoikis. According to this, we also demonstrated that NV669 also downregulated FAK, a non-receptor kinase associated in the adhesion signaling pathway and promote cell detachment. FAK is over-expressed in a variety of human tumors, including PDAC and HCC. Activities of FAK promote tumor initiation, self-renewal, metastasis and regulate cell signaling within the tumor environment in PDAC [75] and HCC [76]. These observations confirm that NV669 could serve as an anticancer reagent in hepatocellular carcinoma and pancreatic cancer. Although, further studies are required to determine the molecular mechanisms by which NV669 alters the FAK and cadherins expression and regulate cell-cell and cell-matrix contacts, it is interesting to notice that inhibition of PTP1B has been previously described to promote cell-cell adhesion disruption and anoikis in breast epithelial cells [24].

In conclusion, the present study is the first to report that NV669, a new derivative of squalamine, has a significant antiproliferative and pro-apoptotic effects against human PDAC and HCC models *in vitro* and *in vivo*. This effect might be related to an inhibition of PTP1B activity. Although further studies are still necessary, the present data suggest that inhibition of PTP1B activity by aminosterol such as NV669 is a promising target for pancreatic and liver cancer therapy.

## MATERIALS AND METHODS

### Chemistry

All solvents were purified according to reported procedures, and the reagents used were commercially available. Methanol, ethyl acetate, dichloromethane were purchased from Sigma and used without further purification. Column chromatography was performed on Merck silica gel (70-230 mesh). ^1^H NMR and ^13^C NMR spectra were recorded in MeOD on a Bruker AC 300 spectrometer working at 300 MHz and 75 MHz, respectively (the usual abbreviations are used: s: singlet, d: doublet, t: triplet, q: quadruplet, m: multiplet). Tetramethylsilane was used as the internal standard. All chemical shifts are given in ppm. Mass spectroscopy analyses have been performed by the Spectropole (Analytical Laboratory) of Aix-Marseille University. The purity of the compounds was checked by analytical HPLC (C18 column, eluent CH_3_CN-water-TFA (90:10:0.025, v/v/v), 0.5-1 mL/Min) with PDA detector spanning from 210 nm to 310 nm. All compounds possessed purity above 95%, as determined by analytical HPLC-PDA at 214 and 254 nm.

### Synthesis of cholest-4-ene-3, 6-dione

In a 250 mL two necked round flask cholesterol (5g, 1.29 10^-2^ mol) was dissolved in anhydrous dichloromethane (50 mL) and pyridinium chlorochromate (8.34 g, 3.87 10^-2^ mol) was added. The mixture was stirred at room temperature for 3 days. Then an additional portion of pyridinium chlorochromate (4.2 g, 1.93 10^-2^ mol) was added. After further stirring at room temperature for 1-day, dry diethylether (150 mL) was added and the liquid was decanted from a brown gum. The insoluble residue was washed three times with dry diethylether (3 × 50 mL). The combined organic layers were washed with water, dried over MgSO_4_, passed through a pad of Florisil and concentrated *in vacuo*. The residue was purified by chromatography on a silicagel column using petroleum ether/ethylacetate (5/2) as eluent affording the expected cholest-4-ene-3, 6-dione **2** in 72% yield.

White solid; mp: 119°C;^ 1^H NMR: δ = 7.15 (s, 1H), 0.55-2.72 (m, 41H); ^13^C: δ = 202.38, 199.53, 161.13, 125.48, 56.59, 56.00, 51.01, 46.85, 42.58, 39.85, 39.50, 36.11, 35.72, 34.25, 34.02, 28.05, 24.01, 23.84, 22.86, 22.60, 20.92, 18.69, 17.55, 11.93. C_27_H_42_O_2_ calcd C 81.4, H 10.6; found C 81.2, H 10.8.

### General procedure for the titanium–mediated reductive amination reaction of cholest-4-ene-3,6-dione

A mixture of cholest-4-ene-3,6-dione **2** (250 mg, 0.63 mmol), titanium(IV) isopropoxide (600 mg, 2.1 mmol) and spermine (404 mg, 2 mmol) in absolute methanol (5 mL) was stirred under argon at room temperature for 12 h. Sodium borohydride (50 mg, 1.3 mmol) was then added at -78°C and the resulting mixture was stirred for an additional 2 hours. The reaction was then quenched by adding water (1 mL) and stirring was maintained at room temperature for 20 minutes. The resulting inorganic precipitate was filtered off over a pad of Celite and washed with methanol and ethyl acetate. The combined organic extracts were dried over Na_2_SO_4_, filtered and concentrated *in vacuo* to afford the expected crude amino derivative which was purified by flash chromatography affording the expected polyamino derivative NV669. Purification by column chromatography (silica gel; CH_2_Cl_2_/ MeOH/ NH_4_OH(32%), 7:3:1) afforded a pale yellow solid in 63% yield; ^1^H NMR (300 MHz, CD_3_OD): *δ* = 5.60 (s, 1H), 4.07-4.04 (d, 1H), 3.11 (m, 1H), 2.68-2.57 (m, 11H), 1.95 (m, 2H), 1.73 (m, 1H), 1.69-1.62 (m, 7H), 1.50-1.40 (m, 9H), 1.38-1.20 (m, 7H), 1.18-0.96 (m, 13H), 0.88-0.86 (m, 4H), 0.83 (s, 3H), 0.80-0.78 (m, 6H), 0.69-0.66 (m, 4H). ^13^C NMR (75 MHz, D_3_OD): *δ* = 149.86, 119.23, 69.37, 68.72, 57.76, 57.50, 56.16, 55.95, 50.56, 45.73, 43.82, 43.32, 41.12, 40.88, 40.70, 39.23, 37.55, 37.28, 35.12, 33.01, 30.27, 29.40, 29.31, 28.27, 27.16, 25.18, 23.49, 23.25, 22.47, 20.55, 19.54,12.74. C_37_H_70_N_4_O MS (ESI^+^) m/z 586.5550 (100%, [M+H]^+^).

### Cell culture

Human pancreatic adenocarcinoma BxPC3 cells were grown in standard culture RPMI 1640 medium. Human pancreatic adenocarcinoma MiaPaCa-2 and hepatocellular carcinoma HepG2 and Huh7 cell were cultured in DMEM medium. All media were supplemented with 10 % fetal bovine serum (FBS) and 2 mM glutamine. Cells lines were maintained at 37°C in a humidified 5% CO_2_ atmosphere.

### Cytotoxicity assays

Cells were seeded onto 96-well plates at a density of 1.5×10^3^cells per well for BxPC3 cells, 0.5×10^3^ cells/well for MiaPaCa-2 cells, 3×10^3^ cells/well for HepG2 cells and 0.85×10^3^cells/well for Huh7 cells, and allowed to adhere and to grow overnight in completed media. The cells were then treated with increasing doses of NV669 for various time period. Growth inhibition of cells was studied by using the crystal violet staining assay as previously described [77, 78]. Cells were washed with Dulbecco’s phosphate buffered saline (DPBS), fixed with 50 μL of 1% glutaraldehyde and rinsed in DPBS, every day for three consecutive days. Subsequently, 50 μL of 0.1% crystal violet in 10 % ethanol was added and rinsed in distilled water after 30 minutes incubation. Stained cells were lysed from the plate with 100 μL sodium dodecyl sulphate 1% and light absorbance of the solution was measured at 590 nm. At least six independent experiments were performed for each condition. The concentrations of NV669 that inhibited 50 % of cell growth (IC_50_) were graphically determined.

### Cell cycle analysis by flow cytometry

After treatment with NV669 for the indicated period, cells were washed with PBS, harvested and centrifuged at 3000 rpm for 3 min to obtain cell pellets. The cells were resuspended in 100 μL PBS and 0.1% Tween 20 (Merck, Steinheim, Germany), fixed with 1 mL of 70 % ethanol and recentrifuged. The cells were incubated with 1mg/mL RNase A (100 μL, Macherey Nagel, Düren, Germany) for 15 min at 37°C. 10 mg/mL propidium iodide (400 μL) was added immediately before analysis. DNA content was measured by flow cytometry (FACScan, Becton Dickinson, New Jersey, USA). Cytogram analysis was performed with Mod Fit software (Becton Dickinson).

### Cell cycle progression analysis by BrdU incorporation

The quantification of cell proliferation based on the measurement of BrdU incorporation during DNA synthesis was measured by using ELISA colorimetric immunoassay (Roche, Mannheim, Germany). Cells grown in a 96-well microplate were pulsed with 10 μM BrdU for 2 hours at 37°C, before or after a NV669 treatment for 24 h. After removing the culture medium and washing, the cells were fixed and the DNA denatured. Cells were incubated with peroxidase-conjugated anti-BrdU antibodies. The substrate solution was then added and the reaction terminated by addition of 0.25N H_2_SO_4_, 100 μl/well. The absorbance was measured at 450 nm and 620 nm as the reference wave length.

### Detection of apoptosis by Annexin-V-FITC/ Propidium iodide double labelling

Surface exposure of phosphatidylserine in apoptotic cells was measured by using Annexin-V-FITC staining (BD Biosciences, Pont de Claix, France). After a 24 h treatment, cells were exposed to Annexin-V-FITC and propidium iodide in the dark at room temperature (25°C) for 15 min before flow cytometry acquisition (FACScan) within 1 hour. Processing of data was carried out with Kaluza software (Beckman Coulter, Brea, USA).

### Western blotting

After a 24 hours incubation with NV669, both cells and culture supernatants (containing floating dead cells) were resuspended for 10 min at 95°C in a lysis buffer [62.5 mmol/L Tris-HCl (pH 6.8), 0.5% SDS, 5 % β-mercaptoethanol, 10% glycerol] and sonicated. Equal amounts of proteins were separated by using 8% to 15% SDS-PAGE and electrotransferred onto a nitrocellulose membrane. Membranes were blocked in 3 % BSA and then probed with different antibodies. The primary antibodies used: anti-cyclin B1, Cdk1, cadherin-1 (CDH-1) and cadherin-2 (CDH-2) were from Abcam (Cambridge, UK). Anti-FAK antibody is supplied by Cell Signaling Technology (Massachusetts, USA). Polyclonal anti-phosphoTyr15-Cdk1 was from Millipore (Billerica, Massachusetts, USA). Anti-polyadenosine-5’-diphosphate-ribose-polymerase-1 (PARP-1) and anti-Caspase-8 antibodies were included in procaspase antibody sampler kit of Cell Signaling Technology or came from Abcam. Anti-cadherin-3 (CDH-3) and β-catenin antibodies were from DB Biosciences and Sigma-Aldrich, respectively. Rabbit polyclonal antibody raised against the N-terminus of PTP1B came from LifeSpan BioSciences (Seattle, USA). Anti-β-actin antibody (mouse monoclonal, Sigma-Aldrich) was used as loading control. Peroxidase-conjugated goat anti-mouse antibodies purchased from (Sigma-Aldrich) were used and enhanced chemiluminescence detection kit (Pharmacia Biosciences, Little Chalfont, UK). Densitometric quantification was done using Image J densitometric software (https://imagej.nih.gov/ij).

### *In vivo* murine cancer model

Animal experiments were carried out in accordance with the French guideline for animal care and the directive 2010/631 EU of the European Parliament and were approved by a local ethical committee. Female immune-deficient NMRI nu/nu (nude) mice were obtained at 6 weeks of age from Janvier Laboratory (Saint Berthevin, France). The cells were harvested by a brief exposure to 0.25 % trypsin and 7 mM EDTA. The cells were washed once in medium with serum to stop trypsin action, counted and resuspended in serum-free medium and matrigel (BD Biosciences) in equal volumes. Nude mice were inoculated subcutaneously with BxPC3 (5 × 10^6^ cells/mouse) or HepG2 (10 × 10^6^) cells, respectively. From the day of inoculation, animals received subcutaneous injection of NV669 (4mg/kg of mouse/day) dissolved in PBS. Control animals were inoculated with identical volume of PBS vector. The dose of NV669 was based on previously toxicity tests performed in immunocompetent mice in our laboratory (data not shown). The mice in both the treatment (n = 10) and control (n = 10) groups were sacrificed by CO2 inhalation and tumors were excised, measured, frozen or fixed in formalin. The body weight was recorded weekly, and tumor volumes were also calculated at the same time using the following equation: tumor volume = lenght × (width)^2^ × π/6.

### Immunohistochemistry

Formalin-fixed, paraffin-embedded sections (5 μm) were stained with anti-Ki-67, anti-PCNA and anti-CD31 rabbit polyclonal antibodies (Abcam, Cambridge, UK) and Vectastain Elite ABC kit (Vector laboratories, Burlingame, USA). Results were expressed as percentage of antigen positive cells ± standard deviation per slide (x 200 magnification). A total of at least 10 randomly selected fields was examined and counted from three tumors of treatment and control groups.

### *In situ* detection of apoptotic cells

Apoptotic cells were detected by terminal deoxynucleotidyl transferase-mediating dUTP nick end labelling (TUNEL) assay (Apoptag plus peroxidase *in situ* apoptosis detection kit from Millipore) following the manufacturer's protocol. The apoptosis was evaluated by counting TUNEL-positive cells (brown-staining) as well as the total number of cells in 18 randomly selected fields in each sample at 200x magnification.

### Phosphatase activity assays

Protein Tyrosine Phosphatase 1B (PTP1B) and T-cell protein tyrosine phosphatase (Tc-PTP) activities were measured by a colorimetric assay using PTP1B Tyrosine Phosphatase drug discovery kit (Enzo Life Sciences, Lausen, Switzerland) according to the manufacturer instructions. The detection of free-phosphate released is based on Malachite green assay. The test is performed in a 96 wells ½ volume microplate in which each well contains a 100 μL reaction in Assay Buffer. 2.5 ng/well of recombinant human enzyme, PTP1B or Tc-PTP (residues 1-322, C-terminal deletion, Enzo Life Sciences), was incubated with a 75 μM final concentration of the phosphopeptide substrate (IR5 insulin receptor β-subunit domain residues 1142-1153, pTyr-1146) and with or without drug for 30 min at 30°C. A phosphate standard curve from 0 to 3.0 nmol of inorganic phosphate dilutions was also prepared. The reactions were terminated by addition of 25 μL of released free-phosphate detection reagent BIOMOL®Red and incubated for 20 min at 30°C to develop color. OD_620 nm_ was read on a microplate reading spectrophotometer (BMG Labtech, Champigny sur Marne, France). OD_620 nm_ readings were converted to nmol of PO4^2-^ with the phosphate standard curve. Three triplicate independent experiments were performed for each condition.

### Cell adherence assay

Cells were seeded in culture dishes, grown to 80 % confluency and treated with IC_50_, 24 h concentration of NV669 or H_2_O as control in media for 6, 15, 24 and 48 h. After incubation, the cell culture medium and two washings of adherent cells with PBS were pooled and centrifuged in PBS (20 min at 2000 rpm). The cell pellet was washed twice with PBS and re-centrifuged. This cell pellet containing floating (or detached cells) was resuspended in RIPA lysis buffer supplemented with both phosphatase and protease inhibitor cocktail (Roche Applied Science, Mannheim, Germany). Meanwhile, the adherent cells were scrapped and further lysed with RIPA buffer supplemented with phosphatase and protease inhibitor cocktail for 30 minutes. The total amounts of proteins (using bicinchoninic acid protein assay kit (Sigma-Aldrich, Saint Louis, USA) were determined in adherent and floating cell lysates. The amount of total protein was representative of the amount of cells in each pellet. The cell adherence was expressed as % of adherent cells reported to total (floating plus adherent) cells in NV669 treated medium versus % of adherent cells reported to total (floating plus adherent) cells in control medium.

### Drugs

NV669 as synthesized here was > 95 % pure and stored in distilled water. Squalamine was prepared according to published procedures [6]. Claramine was synthesized in laboratory [22]. Suramin (BML-KI285) came from Enzo Life Science.

### Statistical analysis

Data are expressed as means ± standard deviation of at least three independent experiments. Differences were determined by Mann and Witney or Student’s t tests when appropriate. A p value of <0.05 was considered as significant.

## Abbreviations

HCC: hepatocellular carcinoma
PDAC: pancreatic adenocarcinoma
PTB1B: protein tyrosine phosphatase 1B
